# Antibody-mediated control of anellovirus infection: evidence from people who inject drugs

**DOI:** 10.1128/jvi.01612-25

**Published:** 2025-12-03

**Authors:** Abraham J. Kandathil, Steven J. Clipman, Raghavendran Anantharam, Dylan Duchen, Andrea L. Cox, H. Benjamin Larman, David L. Thomas

**Affiliations:** 1 Department of Medicine, Johns Hopkins University School of Medicine 1500, Baltimore, Maryland, USA; 2 Department of Pathology, Yale University School of Medicine 156177 , New Haven, Connecticut, USA; 3 Institute for Cell Engineering, Division of Immunology, Department of Pathology, Johns Hopkins University School of Medicine 1500, Baltimore, Maryland, USA; St Jude Children's Research Hospital, Memphis, Tennessee, USA

**Keywords:** plasma virome, anelloviruses, antibodies

## Abstract

Infections with viruses belonging to the family *Anelloviridae* are widespread among humans. Although generally considered a commensal, there is evidence to suggest that these infections may be controlled by host immune responses. However, the mechanism of immune control remains unclear. Previous research has also suggested a possible role of anellovirus capsid spikes in immune evasion. To investigate the role of antibodies in controlling infection, we used AnelloScan to profile plasma collected every 6 months over 2 years from 10 persons who inject drugs (PWID). Participants were selected based on viremia patterns: persistent (*n* = 6) versus intermittent (*n* = 4). Long-read metagenomic sequencing revealed a higher median number of alphatorquevirus (TTV) species in participants with persistent viremia compared to those with intermittent viremia (*P* < 0.0001). AnelloScan detected TTV-specific antibodies among all study participants. No significant differences were observed between the two groups when all antibody-reactive peptides located in the capsid were included. However, among participants with intermittent viremia, antibodies were more frequently reactive to peptides located in the amino acid variable region of the capsid spike domain (*P* = 0.0429). These findings suggest that among PWID, antibodies targeting the sequence variable region of the spike domain appear to be associated with control of anellovirus infection. Additionally, anelloviruses might be susceptible to pre-existing immunity, and the amino acid variable region of the spike protein may play a role in viral infectivity.

**IMPORTANCE:**

Anelloviruses are highly diverse and are recognized as the major component of the blood virome in healthy humans. Despite this, little is known about their interactions with their hosts. In this study, we found that anelloviruses can elicit antibody responses. Notably, antibodies that targeted a sequence variable region on spikes present on viral capsids were associated with truncation of plasma viremia. These data suggest a possible mechanism of immune control of anellovirus infections while also indicating a role of the capsid spikes in viral infectivity.

## INTRODUCTION

Members of the family *Anelloviridae* represent a major component of the blood virome in healthy humans, with infections detected as early as the first few months of life (1). While multiple modes of transmission have been suggested, there is direct evidence of blood-borne transmission among adults (2, 3). Their blood-borne mode of transmission contributes to a higher prevalence in certain populations, such as persons who inject drugs (PWID), due to repeated exposure to blood-borne viruses (4, 5). Longitudinal studies among PWID have shown that anellovirus infections follow a dynamic pattern of clearance and reinfection (4, 6). Higher anellovirus burdens among immunocompromised individuals, such as solid organ transplant recipients, further suggest the possibility of immune control of anellovirus infection (7). However, the mechanisms underlying the control of infection remain poorly understood, and direct evidence of immunity to anellovirus is limited. A cross-sectional study using AnelloScan, a T7 phage library representing peptide sequences of more than 800 human anelloviruses, revealed that many of the peptides are non-reactive (8). A recent cryogenic electron microscopy revealed that amino acid sequences exhibited on the spike domains of anellovirus capsids might contribute to immune evasion (9). Anellovirus capsids are composed of single jelly roll (JR) domains and spike domains (9, 10). Based on amino acid sequence divergence, the spike domain can be divided into JR proximal region (P1) and a JR distal sequence variable region (P2) (9, 10). The latter includes previously reported hypervariable regions (HVRs) that have been suggested to have a role in immune evasion (9, 11).

In this study, we profiled the antibody response in plasma over time to determine the role of antibodies in infection control and compare responses within and outside the spike domain. Based on our previous observation that PWID are at greater risk of acquiring anellovirus infections, we selected longitudinal samples from 10 study participants from our injection drug use cohort with anellovirus infections (4, 12). Each PWID was followed for 2 years and identified as having persistent (*n* = 6) or intermittent (*n* = 4) viremia. Long-read sequencing on the nanopore platform revealed a greater number of anellovirus species among individuals with persistent plasma viremia. Assessing antibody responses using AnelloScan, we observed that antibodies targeting the variable P2 region within the spike domain were associated with intermittent, rather than persistent, viremia. These findings suggest that among PWID, anellovirus infection might elicit neutralizing antibodies that have a role in controlling viremia. These results also demonstrate that the P2 region may contribute to viral infectivity.

## RESULTS

### Persistent anellovirus infections are characterized by higher viral richness

We selected 10 study participants (AS01–AS10) from a Baltimore-based PWID cohort in whom we had previously characterized plasma virome components over time (4). In the persistent group, the median age was 27 years (IQR 10), compared with 28 years (IQR 3) in the intermittent group. Women represented 67% of participants in the persistent group, whereas no women were enrolled in the intermittent group. Regarding race, all participants self-identified as White, except for AS10, who self-identified as Black. Race information was not available for AS06, and age was not available for AS09. Participants were selected based on an initial PCR-based screening to identify individuals with and without circulating anellovirus (13). Anellovirus-specific PCR was used for the initial screening as a targeted approach is more sensitive than metagenomic approaches for detecting low levels of viremia (14, 15). For the present 2-year follow-up study, we included five plasma samples from each participant with a median time interval of 183 days (IQR 116) between two visits. Based on patterns of anellovirus detection over 2 years, participants were categorized into persistent (*n* = 6) and intermittent (*n* = 4) viremia. As PCR provided only genus-level information, visits with detectable anellovirus infections were further characterized using long-read metagenomic sequencing on the nanopore platform (16).

**Fig 1:**
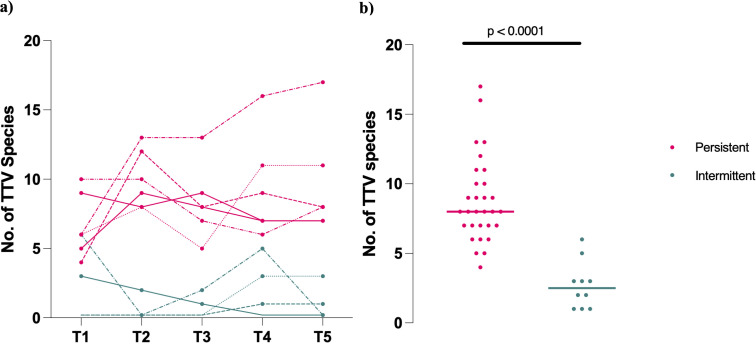
Persistent anellovirus infections are characterized by higher plasma viral richness. In a 2-year longitudinal study involving 10 persons who inject drugs, (**a**) participants with persistent (*n* = 6) viremia exhibited multiple alphatorquevirus (TTV) species at all time points compared to participants with intermittent viremia (*n* = 4). (**b**) Individuals with persistent infection (*n* = 6) had a greater number of TTV species compared to participants with intermittent infection (*n* = 4). Colored horizontal bars denote the median. The *P* values were calculated using a two-tailed Mann-Whitney test. Time points with insufficient TTV reads for species classification were assigned a value of 1.

Species identification of nanopore reads was performed using reference species from the International Committee on the Taxonomy of Viruses (ICTV) Ninth Report (17). Across all sequenced visits, we observed significantly greater alphatorquevirus (TTV) richness, defined as the number of distinct TTV species, among individuals with persistent viremia (*P* < 0.0001, Fig. 1a). We observed a median of 8 (IQR 3.25) TTV species in participants with persistent viremia compared to 2.5 (IQR 2.5) in those with intermittent viremia (Fig. 1b). TTV species dynamics were also different between the two groups (Fig. S1 and S2).

**Fig 2:**
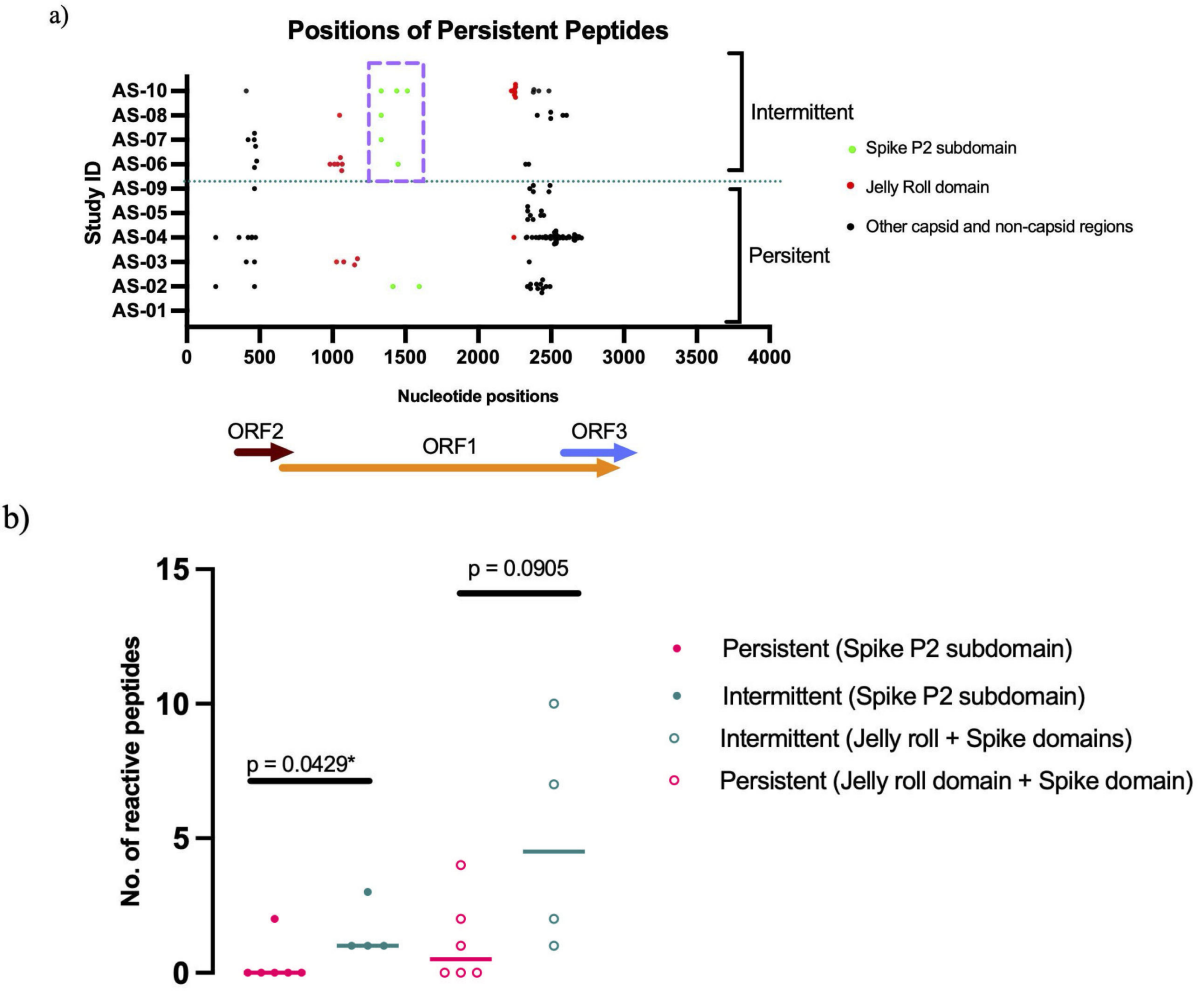
Anellovirus-specific antibodies to amino acid sequence variable region of the spike domain are associated with intermittent anellovirus infection. Testing for anellovirus-specific antibodies was done for 10 persons who inject drugs. (**a**) All participants with intermittent alphatorque viremia (TTV) had persistent antibody reactivity to peptides located in the capsid spike P2 subdomain compared to only one participant with persistent viremia. Participants were longitudinally tested for 2 years. Presence of anellovirus-specific antibodies was assessed using AnelloScan. The capsid protein is encoded by ORF1. Nucleotide positions are as follows: ORF1:589-2898, ORF2:353-712, and ORF3:2567-3074. Peptide positions in the ORF-1-encoded capsid were based on the structural model of TTV21 (GenBank ID: AAK27697) (10). Purple broken lines highlight positions of antibody-reactive peptides in the P2 region among participants with intermittent infection. (**b**) Participants with intermittent anellovirus infections had a higher number of antibodies targeting the spike P2 subdomain. However, no statistical differences were observed between the two groups when looking at the capsid, i.e., jelly roll and spike domains. ORF, open reading frame. Colored horizontal bars denote the median. The *P* values were calculated using a two-tailed Mann-Whitney test.

### Peptide reactivity in the P2 region is associated with control of anellovirus infection

To assess if TTV-specific antibody profiles differed between intermittent and persistent groups, plasma from all time points was tested using the AnelloScan platform (8). AnelloScan is a phage-immunoprecipitation sequencing-based platform to characterize antibody responses to the human anellome (8, 18). The comprehensive phage display library consists of over 32,000 anellovirus peptides representing 829 anellovirus genomes, of which 39.2% represent TTV genomes (8).

Antibodies to TTV peptides were detected at all five time points in all 10 study participants (Fig. S3). However, only a subset of TTV peptides was reactive at all time points. Except for AS01, who lacked peptides consistently reactive across visits, other participants had reactivity to a median of 11 peptides (IQR 10) across visits (Fig. S3). These peptides were distributed throughout the TTV genome (Fig. 2a), and no significant differences were observed between the two viremia groups with respect to the locations of these peptides within the capsid domain (*P* = 0.0905). In contrast, all participants with intermittent viremia exhibited persistent antibody reactivity to at least one TTV peptide between nucleotide positions 1,268–1,673 (P2 spike region) (10), whereas only one participant with persistent viremia showed similar responses in this region (*P* = 0.0429, Fig. 2b). The five study participants who maintained antibody reactivity to the P2 spike region across all time points were observed to have a lower TTV richness (*P* = 0.0003) (Fig. S4a and b). These results suggest that antibody responses targeting the sequence variable P2 region may contribute to controlling anellovirus infection and are more commonly associated with intermittent viremia.

## DISCUSSION

In this investigation, we detected antibodies to TTV in the blood of humans. Moreover, the locus of antibody reactivity was strongly correlated with the outcome of infection. Notably, antibodies to the P2 spike region were associated with viral clearance. These results suggest some of these “commensal” viruses trigger humoral immunity that contributes to infection control and that the P2 region may play a role in viral infectivity.

Reports on immunity against anelloviruses are limited. Indirect evidence suggesting immune control of anelloviruses has been observed in transplant settings, where a higher anellovirus burden is indicative of immunosuppression (5). However, a meta-transcriptomic analysis of human viruses in a variety of tissues in healthy individuals did not detect any upregulated interferon-stimulated gene transcription in tissues containing TTV transcripts (19). A cross-sectional study by Venkataraman et al. using AnelloScan revealed poor antibody reactivity toward most (85%) of the anellovirus peptides, with reactive peptides mostly localized in the C-terminal region of the capsid protein open reading frame 1 (8). The same study showed that among blood transfusion recipients, 11/40 transmitted anelloviruses were associated with antibody response (8). Notably, in all but one of these 11, the earliest antibody reactivities were first detected 100 days post-transfusion, indicating a limited and delayed antibody response (8).

Our data suggest that antibodies targeting the P2 region of the spike domain are associated with viral restriction. This indicates that the residues in the P2 region of the spike domain may be involved in receptor binding and that antibodies might be neutralizing, i.e., interfering with viral entry (20, 21). Receptor-binding regions have been identified in the HVRs of other viruses. For example, the HVR1 of hepatitis C virus (HCV) envelope proteins mediates viral entry and plays a key role in immune evasion (22, 23). Studies in chimpanzees have also demonstrated that antibodies targeting HVR1 help prevent acute HCV infections (23). To evade immune responses, viruses often use sequence variations, such as in the P2 region, to outpace the development of effective immune responses (24). In HCV, antibodies targeting HVR1 have been suggested to drive sequence variation (23). Based on our observations, the P2 region may facilitate immune evasion while also influencing viral infectivity.

This study only included peptides represented in the AnelloScan platform. Additional antibody-reactive peptides might have been missed. In addition, the study likely failed to detect antibodies against conformational, discontinuous, and post-translationally modified epitopes that are not expressed by phage display systems (8). The study was also limited because the onset of exposure (inoculation) of the viruses was not known. Thus, the experience of persons with these viruses prior to our “baseline” was not known. Longer follow-up studies might reveal added insights into the host-virus interactions. Finally, *in vitro* replication models are needed to confirm findings and test hypotheses such as the presence and locus of neutralizing antibodies.

In summary, our data reveal that among PWID, antibodies targeting the sequence variable P2 region of the spike domain are associated with truncation of anellovirus infection and confer protection from infection. These results raise the possibility that susceptibility to anelloviruses varies among individuals and that immune responses may shape the course of infection. Understanding the outcome of these interactions may have broader implications for human health.

## MATERIALS AND METHODS

### Study participants

Study samples were drawn from a cohort of injection drug users located in Baltimore, MD, USA (12). At the time of recruitment, participants reported injecting drugs in the last 6 months and were negative for both HIV and HCV infections. Participants are then longitudinally followed with plasma and serum collected at each monthly visit. For this study, we identified 10 participants with and without plasma anellovirus using a PCR-based screening assay.

### Molecular detection and sequencing

#### DNA extraction

Extraction of plasma RNA and DNA was as previously described (15). Briefly, the Quick-DNA/RNA Miniprep Plus Kit (Zymo Research, USA;Cat #D7003) was used to extract DNA and RNA from 200 µL of plasma. Pre-extraction steps included spinning the samples at 1,600 × *g* for 15 minutes at 4°C to remove debris (e.g., insoluble complexes) followed by filtration using 0.2 µM syringe filters (Thermo Fisher Scientific, USA; Cat #6778-1302).

#### Anellovirus PCR

Previously established protocols were used to detect infection with alpha-, beta-, and gamma-torquevirus (13). Additionally, we enhanced the sensitivity of the PCR by incorporating a rolling circle amplification (RCA) based pre-amplification step, as previously reported (4). As described, RCA was carried out at 30°C for 18 h without the initial 95°C denaturation step using 2 μL of DNA as input. RCA was done using TempliPhi (Sigma Aldrich, USA; Cat #GE25-6400-10). For the PCR, a 1:5 dilution of RCA concatemers was taken for subsequent PCR reactions.

#### Nanopore sequencing

Sequencing for species-level identification of anellovirus infection was done using Oxford Nanopore Sequencing. The RCA concatemers were linearized by an initial debranching step using non-primed Phi29 amplification followed by branch release using S1 endonuclease and a final DNA repair step using a combination of T4 DNA polymerase (Thermo Fisher Scientific, USA) and DNA polymerase I (NEB, USA) (25). Linearized DNA was sheared using a Bioruptor into 1,000–1,500 bp fragments. To obtain an average DNA fragment size of 1,000 bp on the Bioruptor, we used the following settings: 15 seconds on/30 seconds off for two cycles. The size distribution of the sheared DNA samples was visualized using a 2100 Bioanalyzer (Agilent Technologies, USA).

All libraries were prepared using a PCR barcoding protocol (SQK-PBK004, Oxford Nanopore Technologies, UK). Libraries were pooled (*n* = 5) and sequenced on the Oxford Nanopore Technologies GridION platform for 72 h using ~20 to 50 ng of the library on the recommended flow cell. Base calling from the nanopore platform was set to super-accurate mode (> 99% accuracy) with a *Q* score of 10 and no further read filtering.

#### Identification of anellovirus species

We applied our previously described method to identify anellovirus species (16). Briefly, host read deconvolution was done using Minimap2 (26) by mapping them against a custom, selectively masked human reference genome (GRCh38.p14) containing alternate contigs, HLA sequences, and several bacterial contaminant genomes. This masking process prevents anellovirus reads from being inadvertently discarded during host deconvolution (27). Following removal of host sequences, anellovirus species identification was performed by alignment of deconvoluted reads against our in-house database constructed using *Anelloviridae* sequences, as previously described (16). Species identification was based on analysis of ORF1 in its entirety, with a demarcation threshold cutoff of 69% nucleotide similarity (28). A species was considered present if at least five viral reads mapped to the corresponding ICTV reference with mapping quality ≥ 10 and cover ≥ 25% of the reference genome. To further mitigate potential cross-mapping and quantify certainty, we implemented a confidence score that integrates breadth and depth of coverage. Specifically, the score is calculated as:


$$\text{Score}=b_{1\times }\times b_{5\times }\times \log_{10}(1+\bar{d}),$$

where $b_{1\times }$ is the breadth of coverage at ≥1× (fraction of reference positions with depth ≥1), $b_{5\times }$ is the breadth of coverage at ≥5×, and $\bar{d}$ is the mean depth of coverage across the reference genome.

### AnelloScan

The AnelloScan protocol used in this study has been previously described in detail (8). A peptide was defined as reactive when the hits fold change was ≤ 2. Values equal to 1 were classified as unenriched relative to mock immunoprecipitation conditions. For all analyses, only proteins encoded by the sense strand were considered.

### Statistical analyses

All statistical analyses were performed using GraphPad Prism 10 for macOS (version 10.4.2).

A non-parametric statistical test, the Mann-Whitney test, was applied because the data were not normally distributed. Results with a *P* value of <0.05 were deemed significantly different.

## Data Availability

The metagenomic data set generated and analyzed during this study has been deposited in the NCBI Sequence Read Archive with accession code PRJNA1363169.

## SUPPLEMENTAL MATERIAL

Supplemental figures jvi.01612-25-s0001.pdf Figures S1 to S4.

ASM does not own the copyrights to Supplemental Material that may be linked to, or accessed through, an article. The authors have granted ASM a non-exclusive, world-wide license to publish the Supplemental Material files. Please contact the corresponding author directly for reuse.
