# Supplementary material for: Antibody-mediated control of anellovirus infection: evidence from people who inject drugs
Source: J Virol. 2025 Dec 3;99(12):e01612-25. doi: 10.1128/jvi.01612-25 (PMC12724376; doi:10.1128/jvi.01612-25)
Supplement: Supplemental figures — Figures S1 to S4. [file jvi.01612-25-s0001.pdf]

Supplemental Figure 1. Heatmaps of the presence and confidence scores of torque teno virus (TTV) species

a)

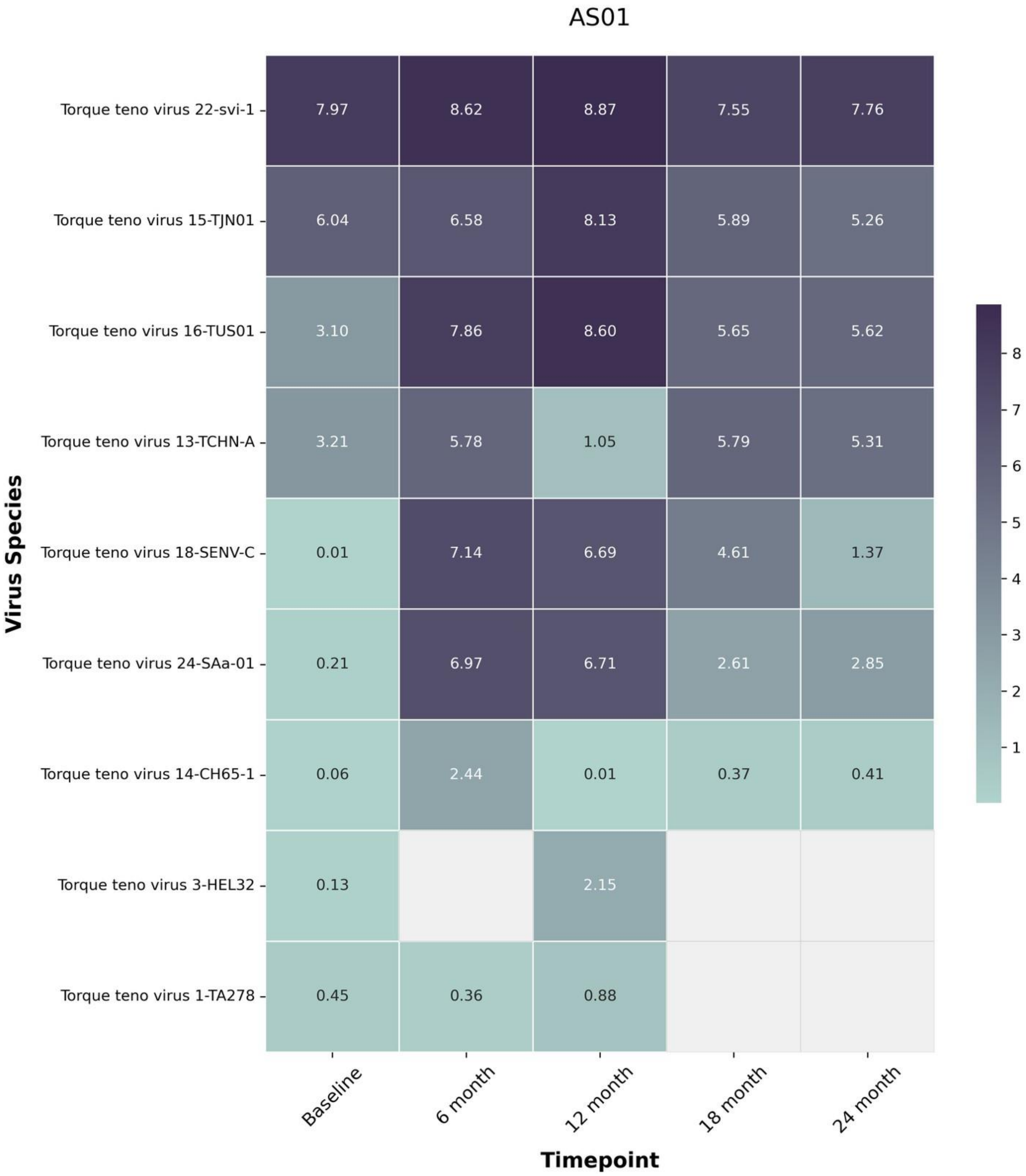

b)

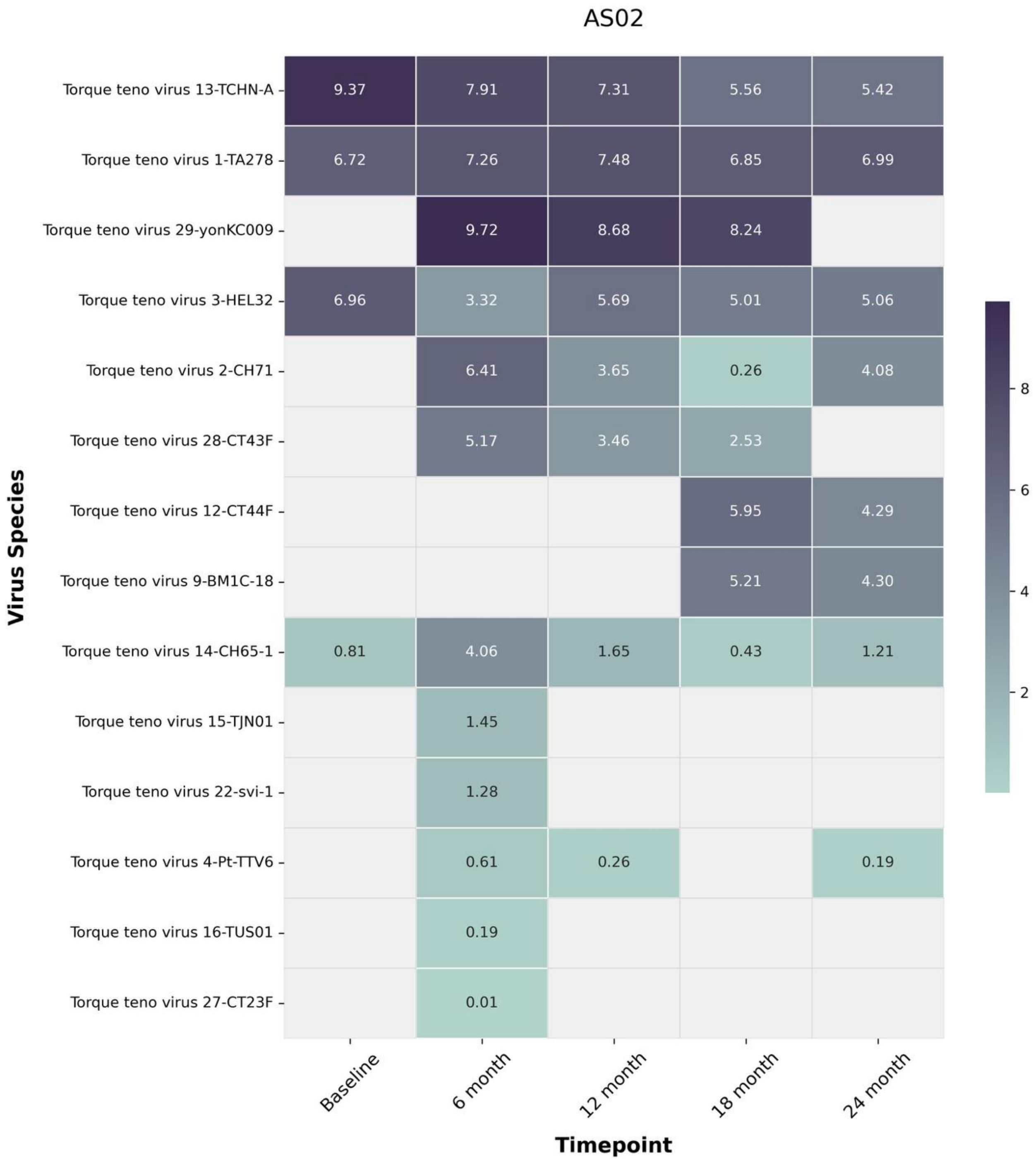
AS02
Virus Species
Timepoint
Torque teno virus 13-TCHN-A
Torque teno virus 1-TA278
Torque teno virus 29-yonKC009
Torque teno virus 3-HEL32
Torque teno virus 2-CH71
Torque teno virus 28-CT43F
Torque teno virus 12-CT44F
Torque teno virus 9-BM1C-18
Torque teno virus 14-CH65-1
Torque teno virus 15-TJN01
Torque teno virus 22-svi-1
Torque teno virus 4-Pt-TTV6
Torque teno virus 16-TUS01
Torque teno virus 27-CT23F
Baseline
6 month
12 month
18 month
24 month
9.37 7.91 7.31 5.56 5.42
6.72 7.26 7.48 6.85 6.99
9.72 8.68 8.24
6.96 3.32 5.69 5.01 5.06
6.41 3.65 0.26 4.08
5.17 3.46 2.53
5.95 4.29
5.21 4.30
0.81 4.06 1.65 0.43 1.21
1.45
1.28
0.61 0.26 0.19
0.19
0.01
8
6
4
2

c)

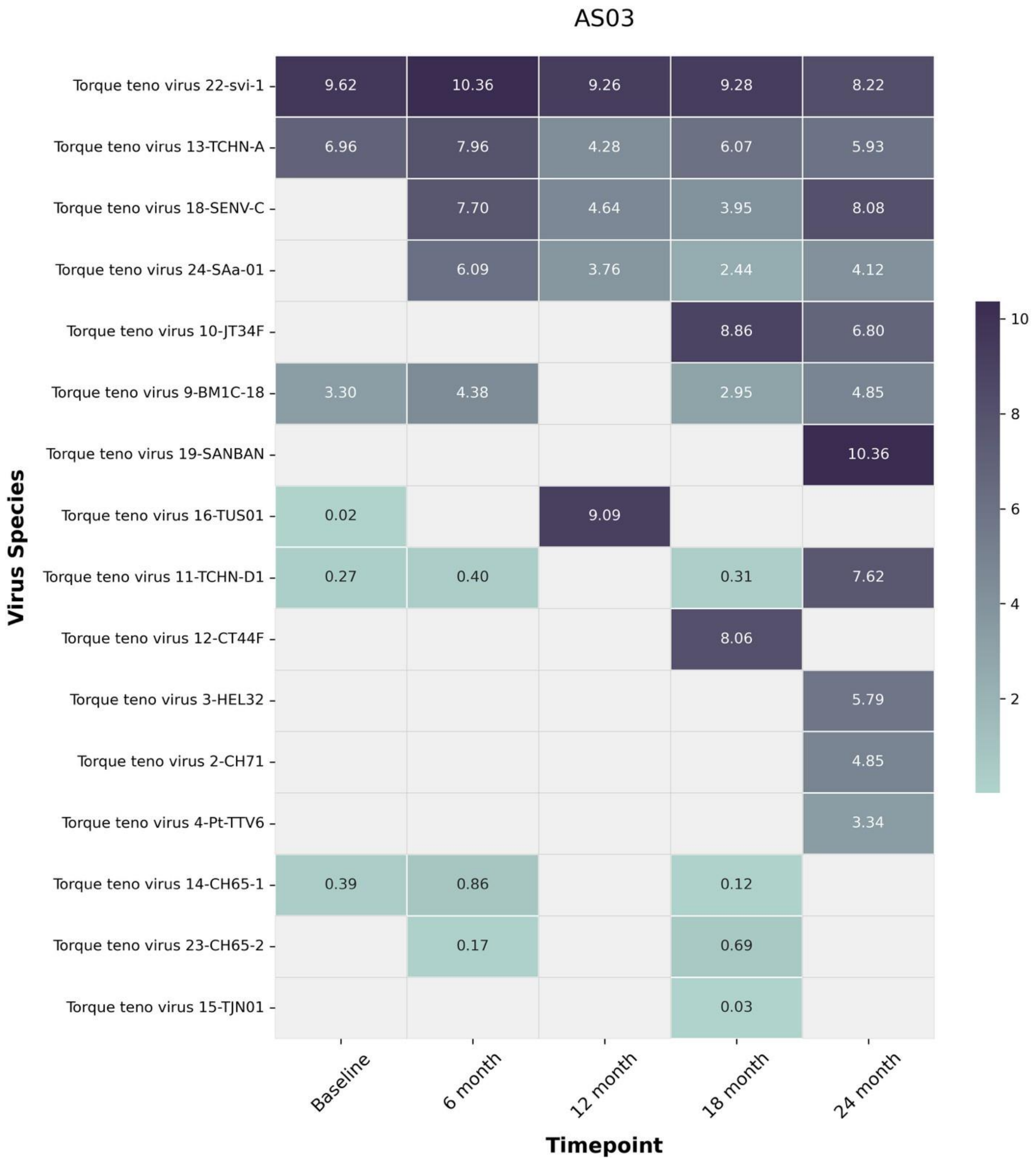

AS03
Torque teno virus 22-svi-1
Torque teno virus 13-TCHN-A
Torque teno virus 18-SENV-C
Torque teno virus 24-SAa-01
Torque teno virus 10-JT34F
Torque teno virus 9-BM1C-18
Torque teno virus 19-SANBAN
Torque teno virus 16-TUS01
Torque teno virus 11-TCHN-D1
Torque teno virus 12-CT44F
Torque teno virus 3-HEL32
Torque teno virus 2-CH71
Torque teno virus 4-Pt-TTV6
Torque teno virus 14-CH65-1
Torque teno virus 23-CH65-2
Torque teno virus 15-TJN01
9.62 10.36 9.26 9.28 8.22
6.96 7.96 4.28 6.07 5.93
7.70 4.64 3.95 8.08
6.09 3.76 2.44 4.12
8.86 6.80
3.30 4.38 2.95 4.85
10.36
0.02 9.09
0.27 0.40 0.31 7.62
8.06
5.79
4.85
3.34
0.39 0.86 0.12
0.17 0.69
0.03
Virus Species
Baseline
6 month
12 month
18 month
24 month
Timepoint
10
8
6
4
2

d)

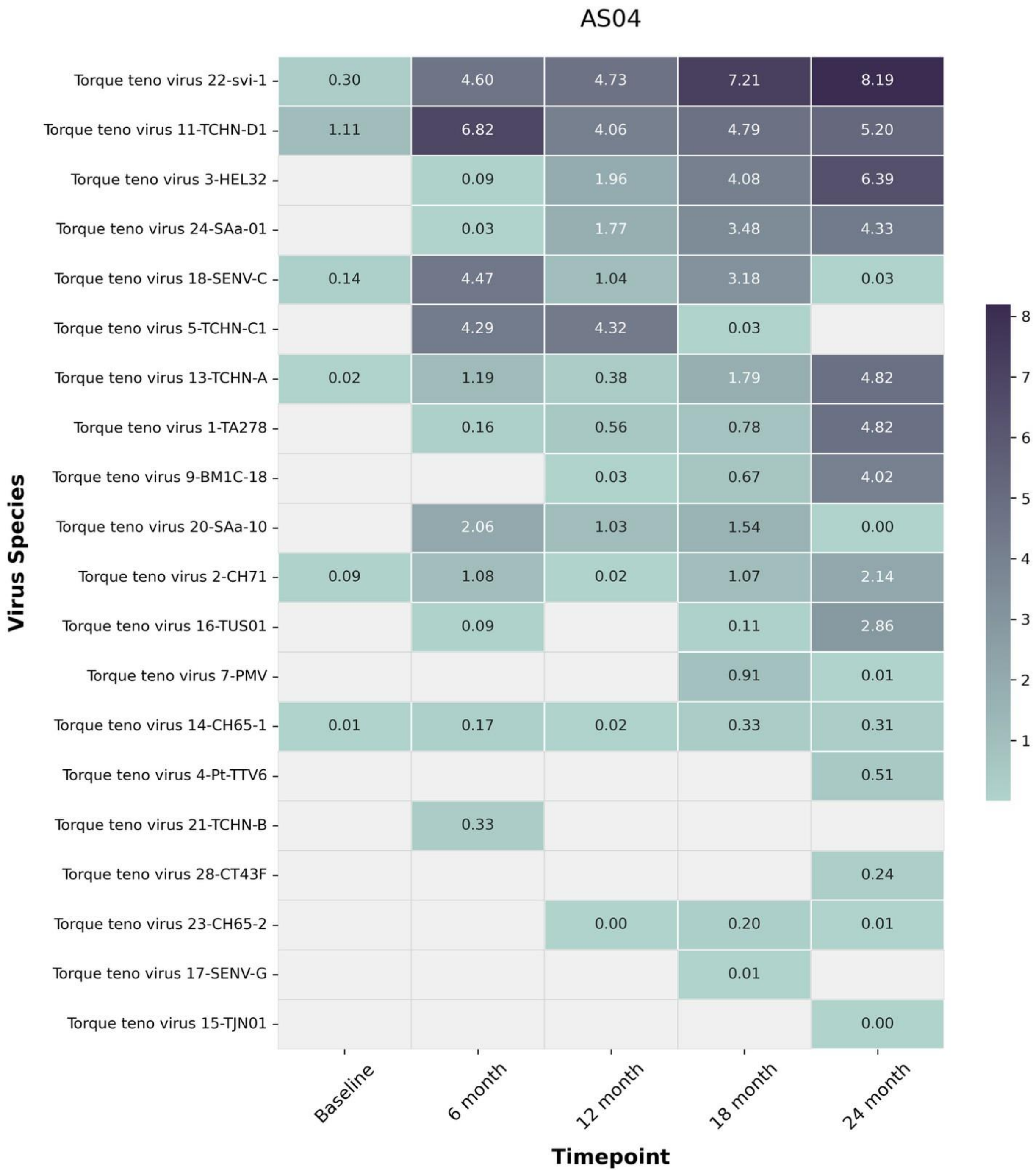

AS04
Virus Species
Timepoint
Baseline
6 month
12 month
18 month
24 month
Torque teno virus 22-svi-1
Torque teno virus 11-TCHN-D1
Torque teno virus 3-HEL32
Torque teno virus 24-SAa-01
Torque teno virus 18-SENV-C
Torque teno virus 5-TCHN-C1
Torque teno virus 13-TCHN-A
Torque teno virus 1-TA278
Torque teno virus 9-BM1C-18
Torque teno virus 20-SAa-10
Torque teno virus 2-CH71
Torque teno virus 16-TUS01
Torque teno virus 7-PMV
Torque teno virus 14-CH65-1
Torque teno virus 4-Pt-TTV6
Torque teno virus 21-TCHN-B
Torque teno virus 28-CT43F
Torque teno virus 23-CH65-2
Torque teno virus 17-SENV-G
Torque teno virus 15-TJN01
0.30 4.60 4.73 7.21 8.19
1.11 6.82 4.06 4.79 5.20
0.09 1.96 4.08 6.39
0.03 1.77 3.48 4.33
0.14 4.47 1.04 3.18 0.03
4.29 4.32 0.03
0.02 1.19 0.38 1.79 4.82
0.16 0.56 0.78 4.82
0.03 0.67 4.02
2.06 1.03 1.54 0.00
0.09 1.08 0.02 1.07 2.14
0.09 0.11 2.86
0.91 0.01
0.01 0.17 0.02 0.33 0.31
0.51
0.33
0.24
0.00 0.20 0.01
0.01
0.00
1
2
3
4
5
6
7
8

e)

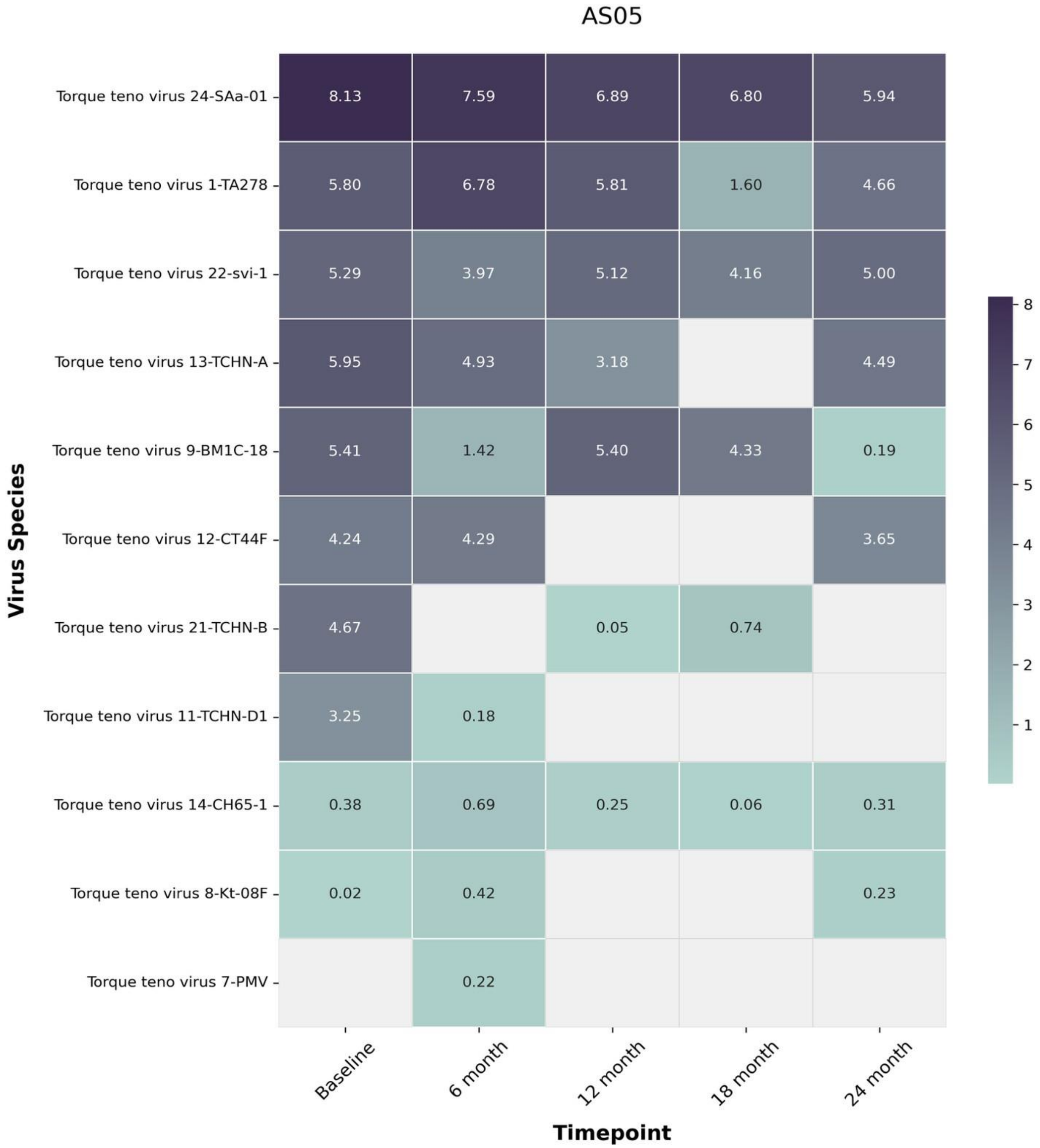
AS05
Torque teno virus 24-SAa-01
Torque teno virus 1-TA278
Torque teno virus 22-svi-1
Torque teno virus 13-TCHN-A
Torque teno virus 9-BM1C-18
Torque teno virus 12-CT44F
Torque teno virus 21-TCHN-B
Torque teno virus 11-TCHN-D1
Torque teno virus 14-CH65-1
Torque teno virus 8-Kt-08F
Torque teno virus 7-PMV
Virus Species
8.13 7.59 6.89 6.80 5.94
5.80 6.78 5.81 1.60 4.66
5.29 3.97 5.12 4.16 5.00
5.95 4.93 3.18 4.49
5.41 1.42 5.40 4.33 0.19
4.24 4.29 3.65
4.67 0.05 0.74
3.25 0.18
0.38 0.69 0.25 0.06 0.31
0.02 0.42 0.23
0.22
Baseline
6 month
12 month
18 month
24 month
Timepoint
8
7
6
5
4
3
2
1

f)

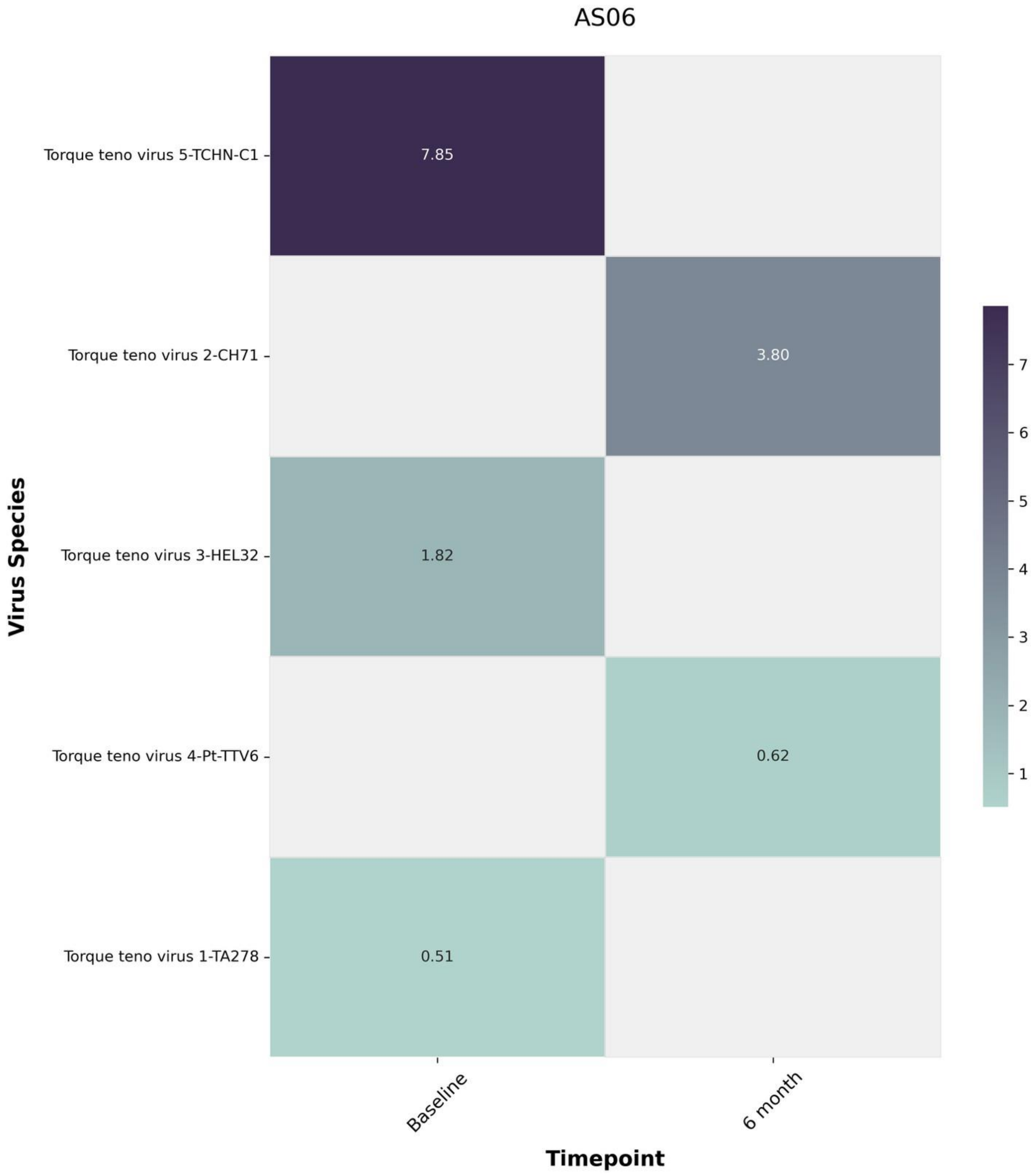
AS06
Virus Species
Torque teno virus 5-TCHN-C1
Torque teno virus 2-CH71
Torque teno virus 3-HEL32
Torque teno virus 4-Pt-TTV6
Torque teno virus 1-TA278
7.85
3.80
1.82
0.62
0.51
Baseline
6 month
Timepoint
1
2
3
4
5
6
7

g)

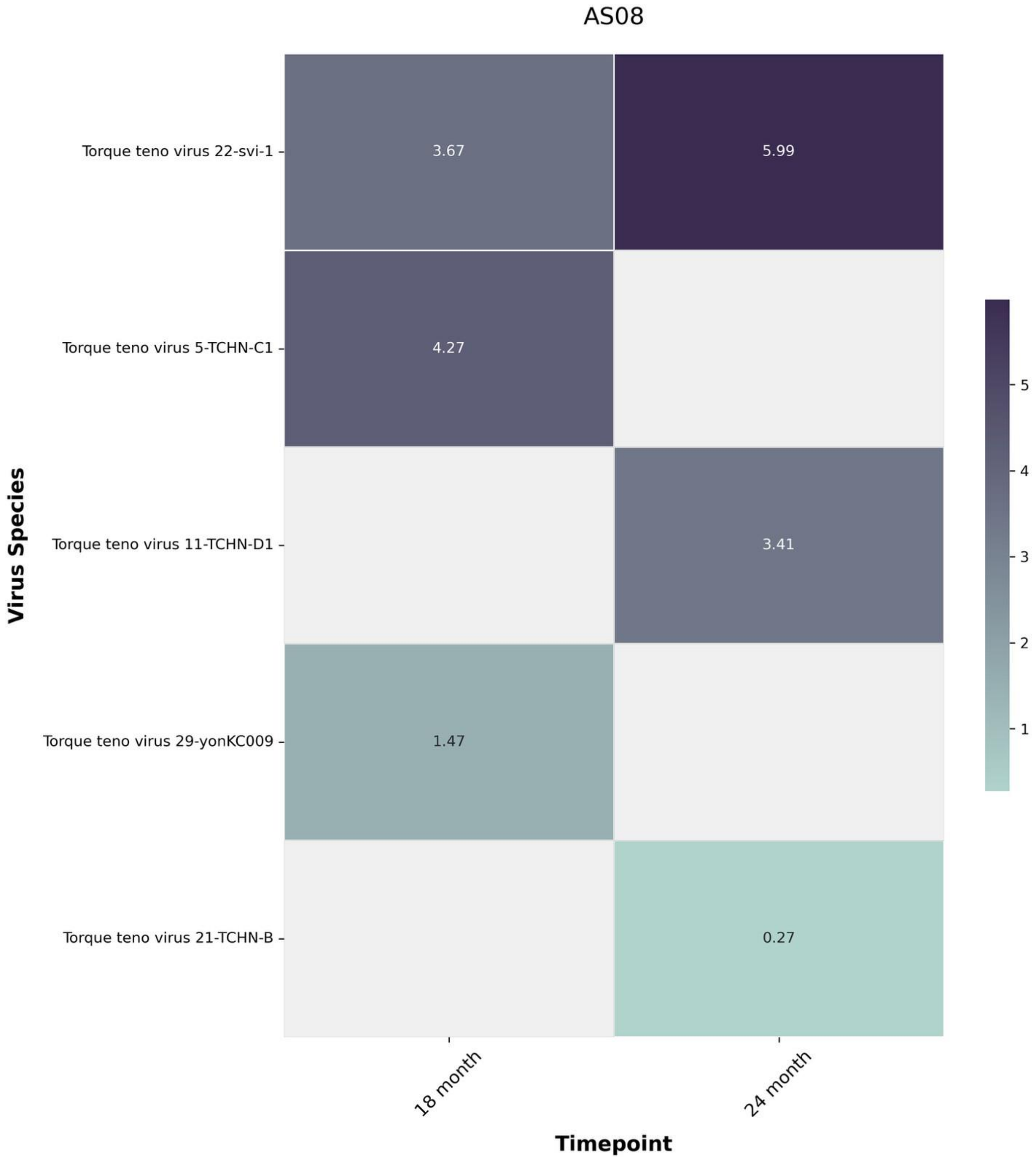

AS08
Torque teno virus 22-svi-1
Torque teno virus 5-TCHN-C1
Torque teno virus 11-TCHN-D1
Torque teno virus 29-yonKC009
Torque teno virus 21-TCHN-B
3.67
5.99
4.27
3.41
1.47
0.27
Virus Species
18 month
24 month
Timepoint
5
4
3
2
1

h)

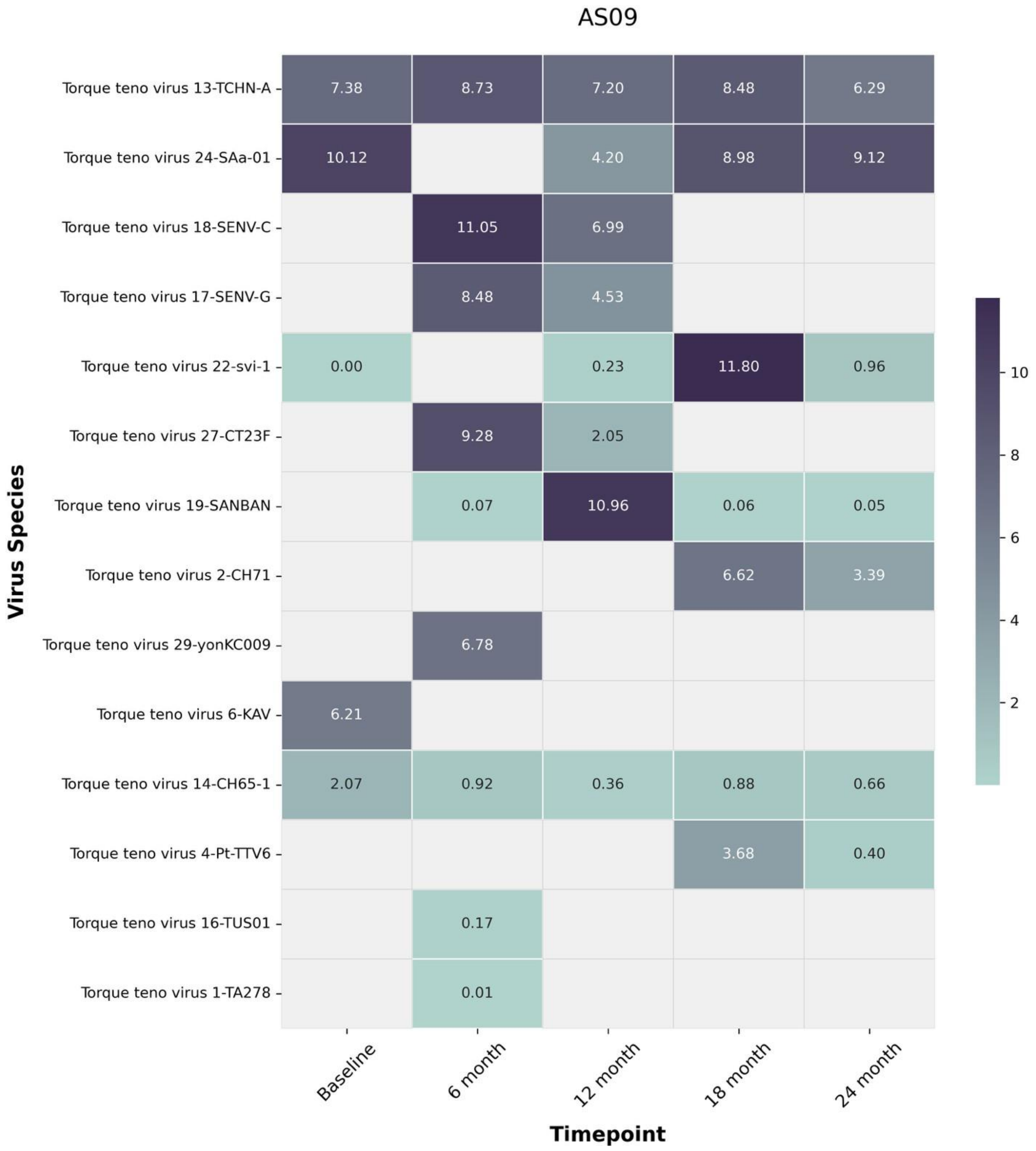
AS09
Torque teno virus 13-TCHN-A
Torque teno virus 24-SAa-01
Torque teno virus 18-SENV-C
Torque teno virus 17-SENV-G
Torque teno virus 22-svi-1
Torque teno virus 27-CT23F
Torque teno virus 19-SANBAN
Torque teno virus 2-CH71
Torque teno virus 29-yonKC009
Torque teno virus 6-KAV
Torque teno virus 14-CH65-1
Torque teno virus 4-Pt-TTV6
Torque teno virus 16-TUS01
Torque teno virus 1-TA278
7.38 8.73 7.20 8.48 6.29
10.12 4.20 8.98 9.12
11.05 6.99
8.48 4.53
0.00 0.23 11.80 0.96
9.28 2.05
0.07 10.96 0.06 0.05
6.62 3.39
6.78
6.21
2.07 0.92 0.36 0.88 0.66
3.68 0.40
0.17
0.01
Virus Species
Baseline
6 month
12 month
18 month
24 month
Timepoint
10
8
6
4
2

i)

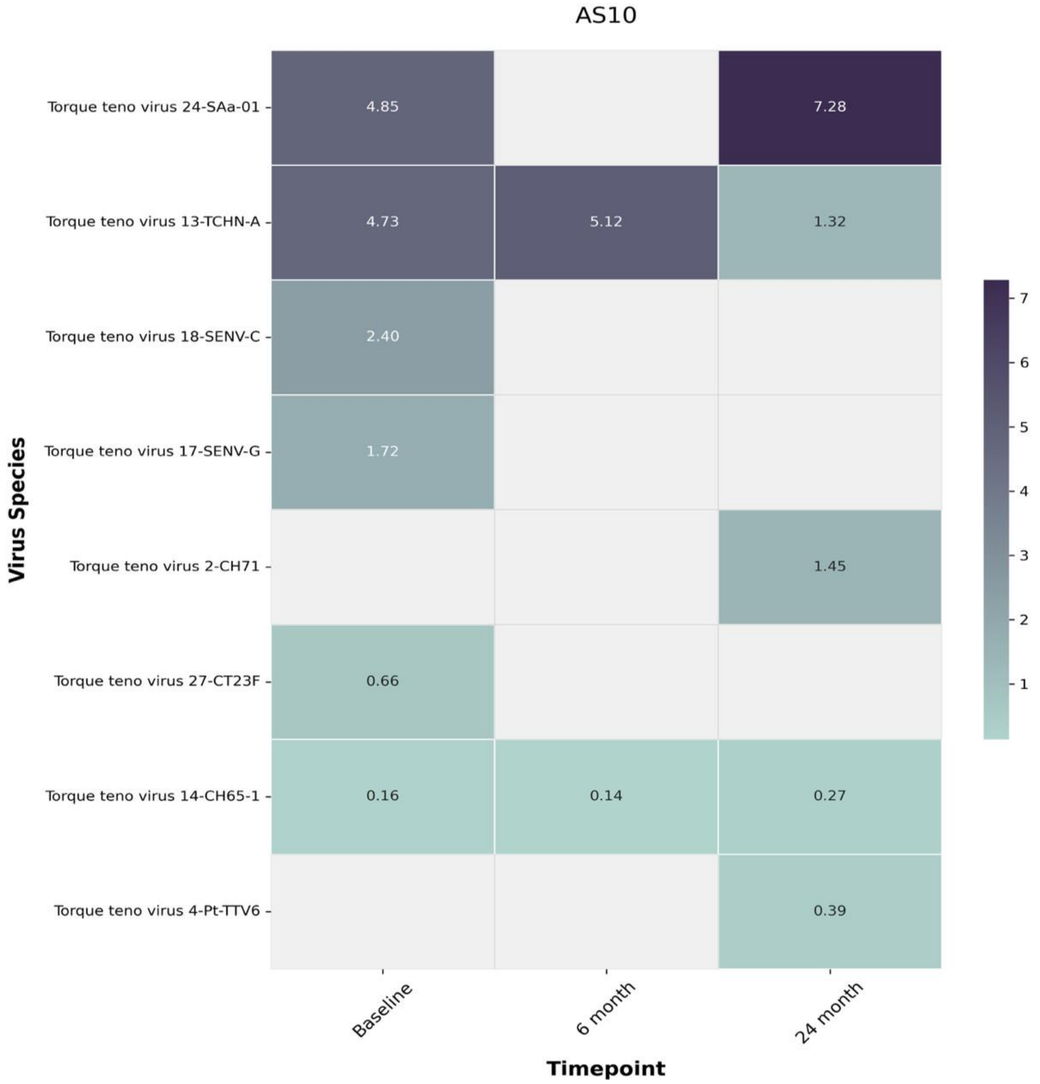

Supplemental Figure 1a-i. Heatmaps of the presence and confidence scores of torque teno virus (TTV) species. Reference species from the International Committee on the Taxonomy of Viruses (ICTV) ninth report (https://ictv.global/report_9th/ssDNA/Anelloviridae) was used to identify species across longitudinal time points for each participant. Each row represents a virus species, and each column a sampling time point. A species was considered present if at least 5 reads mapped with MAPQ ≥ 10 and coverage spanned ≥ 25% of the reference genome. AS07 did not have any ICTV TTV reference species present at this threshold. Color intensity reflects a composite confidence score that combines both breadth and depth of coverage, with greater intensity indicating stronger evidence of presence. This score increases when a larger fraction of the genome is covered and when average depth is higher, while down-weighting spurious low-depth alignments. Gray cells represent the absence of the species at that time point. Species are ordered by their presence patterns, with those consistently detected across all time points appearing first. Cells which are 0.00 but are not grey mean that the score was non-zero (species present) but did not round to 0.01.

Supplemental Figure 2: Higher alphatorquevirus species-specific read counts in individuals with persistent viremia

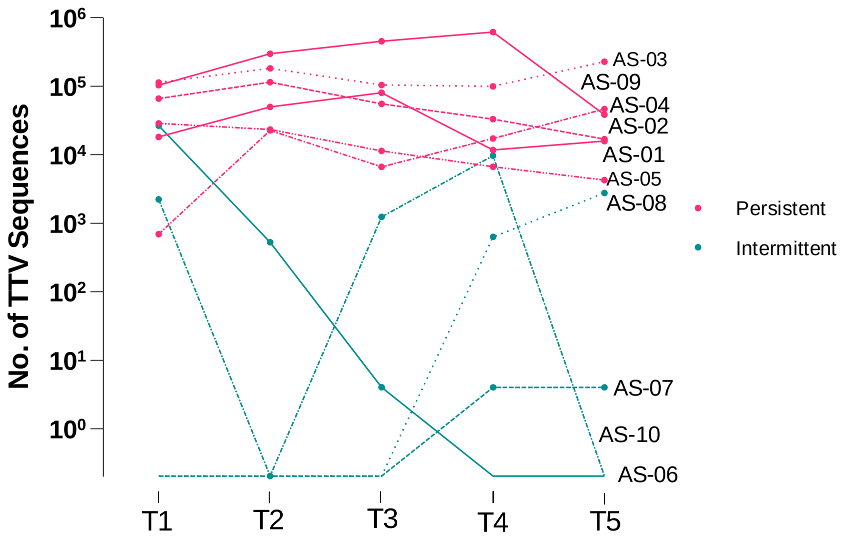

Supplemental Figure 2 : Alphatorquevirus species-specific read counts over time: Alphatorquevirus (TTV) species read counts from 10 study participants (AS01-AS10) profiled at five visits (T1-T5) spaced six months apart using Nanopore sequencing. Participants classified as *intermittent* exhibited undetectable viremia at one or more visits, while those classified as *persistent* had detectable viremia at all visits. Time points without sufficient reads for TTV species classification were assigned a value of 4 for visualization.

Supplemental Figure 3: Reactive AnelloScan peptides and Alphatorquevirus species dynamics over time

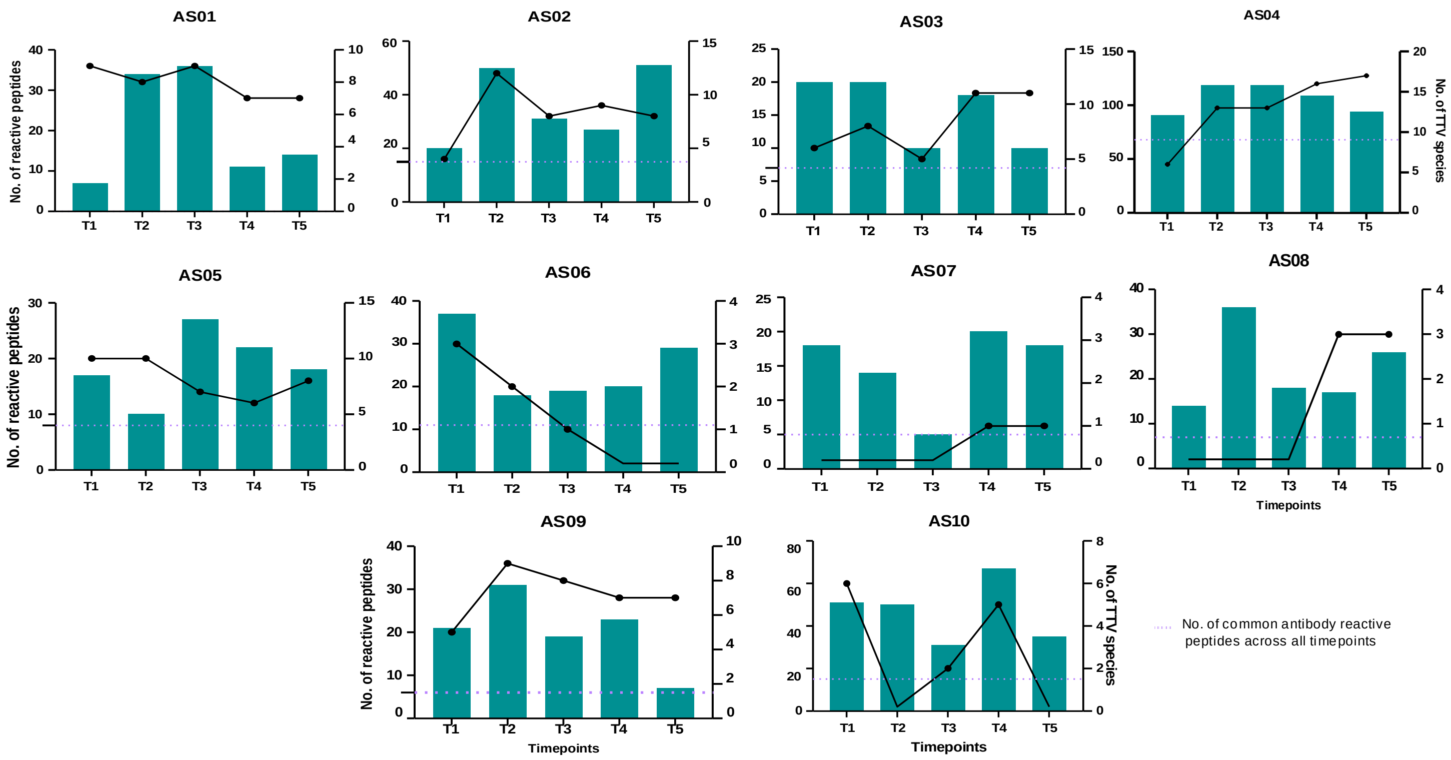

Supplemental Figure 3: **Reactive AnelloScan peptides and Alphatoruqevirus species dynamics over time**: In a two-year longitudinal study done on ten persons who inject drugs, all participants had anellovirus specific antibodies at every time point (T1-T5). Six participants (AS01-AS05, AS09) had persistent plasma viremia while four (AS06-AS08, AS10) had intermittent plasma viremia. Dashed lines indicate number of common alphatoruqevirus (TTV) antibody reactive peptides across all time points for each study participant. One individual (AS01) had no anellovirus specific antibodies that were reactive to same peptide over two years. T1-T5: Denotes time points spanning two years.

Supplemental Figure 4: Lower anellovirus richness in participants who maintained P2-specific antibody responses.

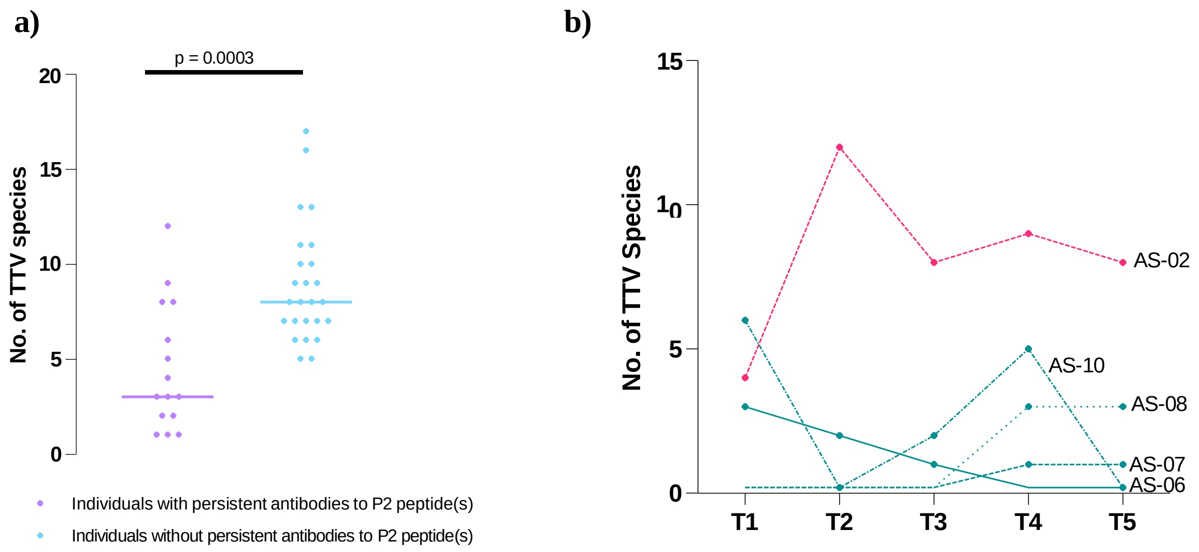

Supplemental Figure 4: Lower anellovirus richness in participants who maintained P2- specific antibody responses: Assessment of antibody responses and alphatorquevirus (TTV) species in plasma collected over two years revealed **a)** Individuals who maintained (n=5) plasma antibodies to spike P2 peptides had a lower number of circulating TTV species compared to individuals who did not maintain P2-specific antibodies (n=5). Colored horizontal bars denote the median. P-values were calculated using a two-tailed Mann-Whitney test. **b)** TTV species dynamics among the five individuals who maintained antibodies to spike P2 peptides in plasma samples over two years revealed clearance and control of plasma viremia.
